# Association of History of Spontaneous or Induced Abortion With Subsequent Risk of Gestational Diabetes

**DOI:** 10.1001/jamanetworkopen.2022.0944

**Published:** 2022-03-03

**Authors:** Yan Zhao, Yongbo Zhao, Kechen Fan, Liping Jin

**Affiliations:** 1 Shanghai Key Laboratory of Maternal Fetal Medicine, Shanghai First Maternity and Infant Hospital, School of Medicine, Tongji University, Shanghai, China

## Abstract

**Importance:**

Gestational diabetes (GD) is one of the most common and important complications of pregnancy. Identifying pregnant women who are at high risk of GD is crucial for implementing early prevention and intervention.

**Objective:**

To examine whether a history of spontaneous abortion (SAB) or induced abortion is associated with increased risk of GD in subsequent pregnancies.

**Design, Setting, and Participants:**

This retrospective cohort study was conducted at a tertiary hospital in Shanghai, China. Pregnant women who received routine antenatal care between January 2014 and December 2019 were included. Data analysis was performed from December 2020 to June 2021.

**Exposures:**

Maternal history of abortion, including SAB and induced abortion, were extracted from medical records.

**Main Outcomes and Measures:**

GD was diagnosed with a 75-g diagnostic oral glucose tolerance test. A multivariable-adjusted log-binomial analysis was used to estimate relative risks (RRs) and 95% CIs of GD associated with history of abortion.

**Results:**

Among the 102 259 included pregnant women (mean [SD] age, 29.8 [3.8] years), 14 579 (14.3%) experienced only SAB, 17 935 (17.5%) experienced only induced abortion, and 4017 (3.9%) experienced both SAB and induced abortion. A total of 12 153 GD cases were identified, and the prevalence of GD was 11.9% (12 153 of 102 259 women) in this cohort. Pregnant women who experienced only SAB (RR, 1.25; 95% CI, 1.18-1.31) or both SAB and induced abortion (RR, 1.15; 95% CI, 1.05-1.27) were at higher risk of developing GD. The association of SAB history with GD occurred in a number-dependent manner. Compared with pregnant women with no history of abortion, the RR for GD increased by 18% (RR, 1.18; 95% CI, 1.11-1.26) for pregnant women with 1 SAB, by 41% (RR, 1.41; 95% CI, 1.27-1.57) for those with 2 SABs, and by 43% (RR, 1.43; 95% CI, 1.22-1.67) for those more than 2 SABs. However, no association between history of induced abortion and GD was observed.

**Conclusions and Relevance:**

This study found that a history of SAB was associated with increased risk of GD in subsequent pregnancies. Further research is needed to clarify this association and explore the potential biological mechanisms underlying the association.

## Key Points

**Question:**

Is a history of spontaneous abortion (SAB) or induced abortion associated with increased risk of gestational diabetes (GD) in subsequent pregnancies?

**Findings:**

In this cohort study of 102 259 Chinese pregnant women, a history of SAB was significantly associated with increased risk of GD in a number-dependent manner.

**Meaning:**

These findings suggest that pregnant women with a history of SAB, especially those with a history of recurrent SAB, should pay particular attention to monitoring their blood glucose and implementing early prevention of and intervention for GD.

## Teaser

This cohort study examines whether a history of spontaneous or induced abortion is associated with increased risk of gestational diabetes in subsequent pregnancies.

## Introduction

Gestational diabetes (GD) is one of the most common and important complication of pregnancy, affecting 7% to 25% of all clinically recognized pregnancies worldwide.^1,2,3^ The high prevalence of GD has raised substantial concerns because it is associated not only with adverse perinatal outcomes but also with increased long-term cardiovascular and metabolic health risk in both mothers and their offspring.^4,5,6,7,8^ It is, therefore, crucial to identify pregnant women who are at high risk of GD and to implement early monitoring and intervention.

Abortion, which may occur spontaneously or intentionally, is common globally.^9^ It is estimated that up to 30% of all pregnancies terminate in spontaneous abortion (SAB),^10,11^ and more than 43 million elective induced abortions occur annually worldwide.^12^ Studies have shown that SAB, especially recurrent SAB, is associated with later maternal risk of cardiovascular disease and venous thromboembolism,^13,14,15,16^ and women with a history of induced abortion are at higher risk of developing metabolic diseases, including type 2 diabetes, later in life.^17,18,19^ The pathophysiological mechanisms for the elevated cardiovascular and metabolic disease risk might be associated with oxidative stress and inflammation,^18,20^ which also contribute to the development of GD.^21,22,23^ However, whether prior abortion would be associated with increased maternal risk of GD in subsequent pregnancies remains unclear.

The aim of the present study was to examine the association between abortion history and the subsequent risk of GD among pregnant Chinese women. Furthermore, considering that induced abortion and SAB are different biological events, we also examined whether different types of abortion had different associations with GD risk.

## Methods

### Study Participants

This retrospective cohort study was conducted at a tertiary care hospital that is one of the largest prenatal health care providers in Shanghai, China, with more than 45 000 inpatients per year. The hospital’s institutional review board approved this study and waived the requirement for informed consent because all data were anonymized. This study follows the Strengthening the Reporting of Observational Studies in Epidemiology (STROBE) reporting guideline.

**Figure 1.**
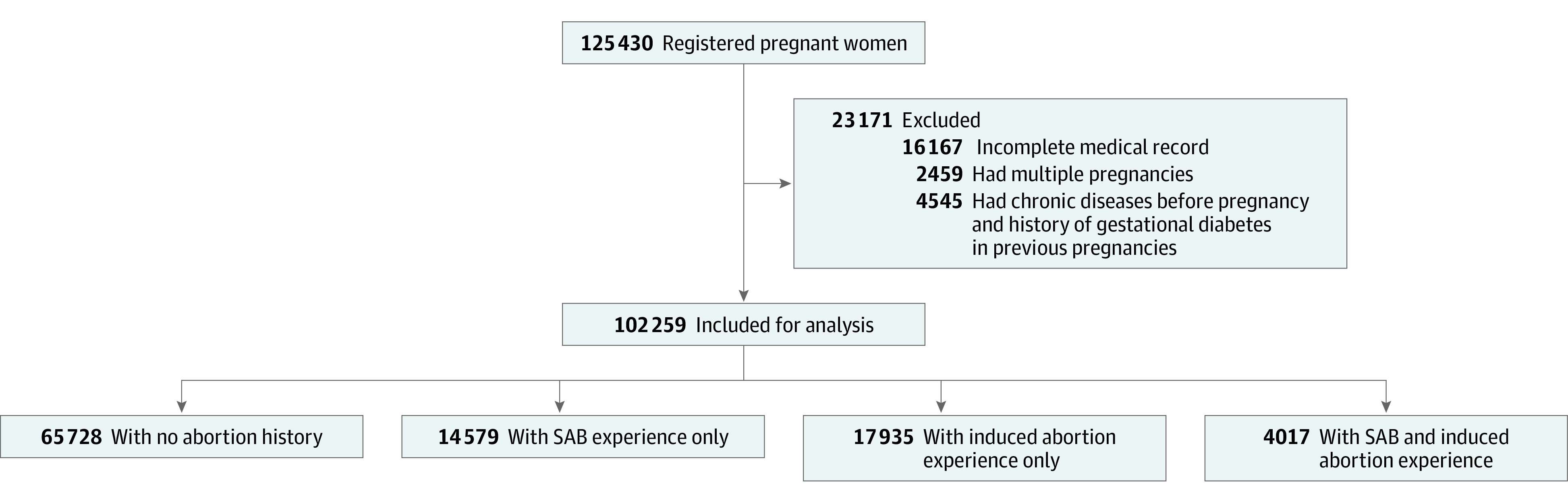
Participant Enrollment Flowchart A total of 102 259 pregnant women were included in the final analysis: 14 579 (14.3%) experienced only spontaneous abortion (SAB), 17 935 (17.5%) experienced only induced abortion, and 4017 (3.9%) experienced both SAB and induced abortion.

Participants were pregnant women who registered in the outpatient clinics of the department of obstetrics and visited the clinic regularly from January 2014 to December 2019. The flowchart of participant enrollment is presented in Figure 1. Briefly, the medical records of 125 430 pregnant women were reviewed, and those who had complete medical records during pregnancy and underwent a standard oral glucose tolerance test (OGTT) at 24 to 28 gestational weeks were included (109 263 women). Women with multiple pregnancies (2459 women), a history of GD in previous pregnancies, and a history of chronic diseases (hypertension, diabetes, kidney disease, and thyroid dysfunction) before this pregnancy were further excluded (4545 women).

### Exposure

During the first prenatal visit, all participants were interviewed by a trained nurse. Reproductive history was collected and recorded in the electronic medical record system of the hospital. Abortion data, including SAB and induced abortion, were extracted via manual review of the electronic medical record. Participating pregnant women were categorized as exposed if they experienced only SAB, only induced abortion, or both SAB and induced abortion.

### Clinical Variables

Demographic characteristics such as maternal age, parity, age at menarche, body weight and height before pregnancy, family history of diabetes, and the use of assisted reproduction technology (ART) during this pregnancy were also extracted from the medical record system of the hospital. Prepregnant body mass index was calculated as body weight in kilograms divided by height in meters squared.

### Outcome

The outcome of interest was GD, which was screened for and diagnosed at 24 to 28 weeks of gestation using the OGTT according to the recommendations of the International Association of Diabetes and Pregnancy Study Groups.^24^ For the test, pregnant women were required to fast overnight and then were given 75 g of glucose by oral ingestion in the morning. The fasting blood glucose (FBG) levels and blood glucose levels 1 and 2 hours after glucose ingestion were measured. GD was diagnosed according to the following criteria: FBG levels greater than or equal to 92 mg/dL (to convert to millimoles per liter, multiply by 0.0555), 1-hour blood glucose levels greater than or equal to 180 mg/dL, or 2-hour blood glucose levels greater than or equal to 153 mg/dL.

### Statistical Analysis

All participants were categorized into 4 groups: no abortion group (pregnant women who did not experience SAB or induced abortion), SAB only group (pregnant women who experienced only SAB), induced abortion only group (pregnant women who experienced only induced abortion), and both SAB and induced abortion group (pregnant women who experienced both SAB and induced abortion). The general characteristics of the participants are presented as numbers and percentages, and nonnormal distributed glucose levels are presented as medians (IQRs) according to different exposure groups.

Log-binomial regression analysis was performed to estimate the relative risk (RR) and 95% CI for the associations of different types of abortion with incident GD. In log-binomial analysis, the no abortion group served as the reference. Potential confounders, including maternal age (continuous), maternal parity (nulliparous or multiparous), age at menarche (continuous), family history of diabetes (yes or no), the use of ART (yes or no), and prepregnancy body mass index (continuous), were adjusted in the log-binomial analyses. Those confounders were selected on the basis of prior knowledge of their associations with abortion or GD and the data accessibility.

In a secondary analysis, the SAB only group was further divided into participants with 1, 2, and more than 2 SABs groups. The association between the number of SABs and GD risk was estimated by log-binomial analysis with the no abortion group serving as the reference. This log-binomial analysis adjusted for the aforementioned confounders.

As a sensitivity analysis to address the potential selection bias, we replicated the log-binomial analysis by restricting the participants to nulliparous pregnant women. Another sensitivity analysis was performed to exclude the association of a family history of diabetes by restricting the participants to pregnant women without a family history of diabetes. In the third sensitivity analysis, we replicated the log-binomial analysis to participants who conceived spontaneously to ensure that the observed associations were not confounded by the use of ART. All statistical analyses were performed using R statistical software version 3.2.3 (R Project for Statistical Computing), and 2-sided *P* < .05 was considered significant. Data analysis was performed from December 2020 to June 2021.

## Results

### Demographic Characteristics of the Participants

Among the 102 259 included pregnant women (mean [SD] age, 29.8 [3.8] years), 14 579 (14.3%) experienced only SAB, 17 935 (17.5%) experienced only induced abortion, and 4017 (3.9%) experienced both SAB and induced abortion. The general characteristics of the participants according to different exposure groups are summarized in Table 1. Compared with women with no abortion history, those with abortion history were more likely to be multiparous and to have obesity. In addition, the proportion of pregnant women older than 35 years was more than 2-fold higher among pregnant women with abortion history than those with no abortion history.

**Table 1.** General Characteristics of the Pregnant Women According to History of Abortion

Characteristics	Women, No. (%)				
	Total (N = 102 259)	No abortion history (n = 65 728	History of abortion		
			SAB only (n = 14 579)	Induced abortion only (n = 17 935)	Both SAB and induced abortion (n = 4017)
Maternal age, y					
<35	90 099 (88.1)	60 651 (92.3)	12 030 (82.5)	14 561 (81.2)	2857 (71.1)
≥35	12 160 (11.9)	5077 (7.7)	2549 (17.5)	3374 (18.8)	1160 (28.9)
Parity					
Nulliparous	79 695 (77.9)	55 384 (84.3)	11 653 (79.9)	10 369 (57.8)	2289 (57.0)
Multiparous	22 564 (22.1)	10 344 (15.7)	2926 (20.1)	7566 (42.2)	1728 (43.0)
Age at menarche, y					
≤12	21 362 (20.9)	14 203 (21.6)	3092 (21.2)	3354 (18.7)	713 (17.7)
13-14	59 235 (57.9)	38 331 (58.3)	8458 (58.0)	10 166 (56.7)	2280 (56.8)
≥15	21 662 (21.2)	13 194 (20.1)	3029 (20.8)	4415 (24.6)	1024 (25.5)
Prepregnancy body mass index^a^					
<18.5	13 129 (12.8)	9015 (13.7)	1418 (9.7)	2315 (12.9)	381 (9.5)
18.5-24.9	81 605 (79.8)	52 406 (79.7)	11 784 (80.8)	14 170 (79.0)	3245 (80.8)
≥25	7525 (7.4)	4307 (6.6)	1377 (9.4)	1450 (8.1)	391 (9.7)
Use of assisted reproductive technology					
Yes	1535 (1.5)	848 (1.3)	450 (3.1)	131 (0.7)	106 (2.6)
No	10 0724 (98.5)	64 880 (98.7)	14 129 (96.9)	17 804 (99.3)	3911 (97.4)
Family history of diabetes					
Yes	5103 (5.0)	3116 (4.7)	875 (6.0)	883 (4.9)	229 (5.7)
No	97 156 (95.0)	62 612 (95.3)	13 704 (94.0)	17 052 (95.1)	3788 (94.3)

Abbreviation: SAB, spontaneous abortion.

^a^ Body mass index is calculated as weight in kilograms divided by height in meters squared.

### Distributions of Plasma Glucose Levels

FBG and blood glucose levels 1 and 2 hours after glucose ingestion were measured. The median (IQR) blood glucose levels during the OGTT of the 102 259 pregnant women were 77 (76-83) mg/dL for FBG, 133 (115-153) mg/dL at 1 hour, and 115 (101-130) mg/dL at 2 hours (Table 2). On the basis of the OGTT, we identified 12 153 GD cases among 102 259 women, for a GD prevalence of 11.9% in this cohort. The corresponding prevalence of GD was 10.7% (7018 of 65 728 women) in the no abortion history group, 15.7% (2282 of 14 579 women) in the SAB only group, 12.3% (2213 of 17 935 women) in the induced abortion only group, and 15.9% (640 of 4017 women) in the both SAB and induced abortion group (Table 3).

**Table 2.** Distribution of Plasma Glucose Levels at 24 to 28 Gestational Weeks by History of Abortion

Test	Plasma glucose levels, median (IQR), mg/dL				
	Total (N = 102 259)	No abortion history (n = 65 728)	History of abortion		
			SAB only (n = 14 579)	Induced abortion only (n = 17 935)	Both SAB and induced abortion (n = 4017)
Fasting blood glucose	77 (76-83)	77 (74-83)	79 (76-83)	79 (76-83)	79 (76-83)
Oral glucose tolerance test					
1 h	133 (115-153)	133 (115-151)	139 (121-159)	133 (115-153)	139 (119-157)
2 h	115 (101-130)	114 (101-130)	119 (105-135)	114 (101-130)	117 (103-133)

Abbreviation: SAB, spontaneous abortion.

SI conversion factor: To convert plasma glucose level to millimoles per liter, multiply by 0.0555.

**Table 3.** Association of Incident GD With History of Abortion

Abortion history	GD prevalence, No. of women/total No. (%)	RR (95% CI)	
		Crude model	Adjusted model^a^
No abortion history	7018/65 728 (10.7)	1.00 [Reference]	1.00 [Reference]
SAB only	2282/14 579 (15.7)	1.56 (1.48-1.63)^b^	1.25 (1.18-1.31)^b^
Induced abortion	2213/17 935 (12.3)	1.18 (1.12-1.24)^b^	1.04 (0.98-1.10)
Both SAB and induced abortion	640/4017 (15.9)	1.56 (1.45-1.73)^b^	1.15 (1.05-1.27)^b^

Abbreviations: GD, gestational diabetes; RR, relative risk; SAB, spontaneous abortion.

^a^ Adjusted for maternal age, parity, age at menarche, family diabetes history, the use of assisted reproductive technology, and prepregnancy body mass index.

^b^ *P* < .01.

### Associations Between Abortion History and Risk of GD

The associations between abortion history and GD are presented in Table 3. In both crude and adjusted models, a history of abortion was associated with increased risk of GD in subsequent pregnancies. The associations differed according to the type of abortion. Compared with pregnant women with no abortion history, the risk of GD increased by 25% (RR, 1.25; 95% CI, 1.18-1.31) for pregnant women who experienced only SAB and by 15% (RR, 1.15; 95% CI, 1.05-1.27) for pregnant women who experienced both SAB and induced abortion. However, no associations were found for induced abortion.

**Figure 2.**
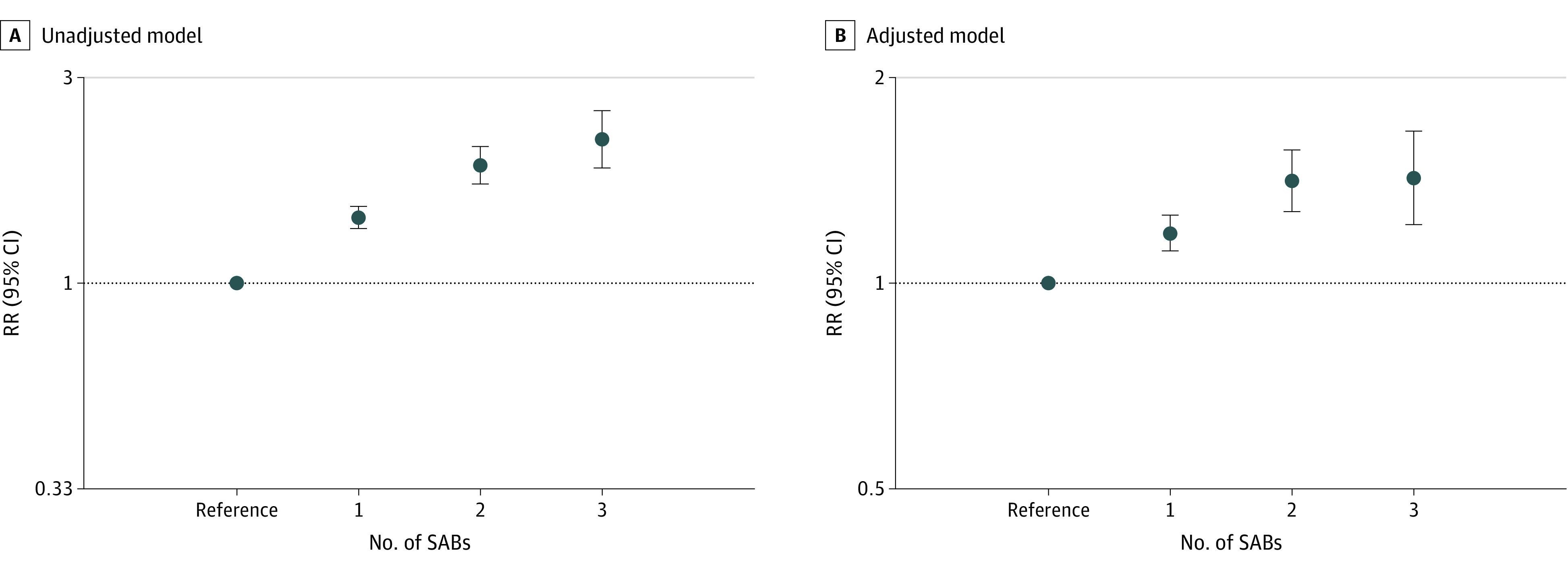
Associations Between the Number of Spontaneous Abortions (SABs) and Gestational Diabetes Risk Graphs show relative risks (RRs) and 95% CIs for the crude model (A) and adjusted model (B), which was adjusted for maternal age, parity, age at menarche, family history of diabetes, the use of assisted reproductive technology, and prepregnancy body mass index.

Because we found that a history of SAB was associated with increased risk of GD, we further explored the association between the number of SABs and GD risk. As shown in Figure 2, positive associations were observed between the number of SABs and the risk of GD, with a monotonic increase in RR. In the adjusted model, compared with pregnant women with no abortion history, pregnant women with 1 SAB had an RR for GD that was increased by 18% (RR, 1.18; 95% CI, 1.11-1.26), those with 2 SABs had an RR that was increased by 41% (RR, 1.41; 95% CI, 1.27-1.57), and those with more than 2 SABs had an RR that was increased by 43% (RR, 1.43; 95% CI, 1.22-1.67) (Figure 2B).

### Sensitivity Analysis

In sensitivity analysis, when restricting the log-binomial analysis to nulliparous pregnant women, the associations between abortion history and GD were similar to those observed in all participating pregnant women (eTable 1 in the Supplement). Furthermore, after restricting the log-binomial analysis to pregnant women without a family history of diabetes or pregnant women who conceived spontaneously, the observed associations also did not appreciably change (eTable 2 and eTable 3 in the Supplement).

## Discussion

This large cohort study of 102 259 pregnant Chinese women provides a comprehensive assessment of the association between history of abortion and the risk of GD in subsequent pregnancies. We found that a history of SAB was associated with increased risk of GD, and these associations occurred in a number-dependent manner. However, there was no association between induced abortion and GD. To our knowledge, this is one of the largest studies to explore such associations in humans.

SAB is one of the most common adverse outcomes in early pregnancy.^25,26^ We are aware of one previous study^27^ that examined the association between a history of SAB and GD in subsequent pregnancies among 16 286 pregnant women from the China National Gestational Diabetes Survey. In line with our study, Yang et al^27^ found that a history of SAB was significantly associated with increased risk of GD, and the adjusted odds ratio was 1.46 (95% CI, 1.12-1.91), which was slightly higher than the RR found in our study (RR, 1.25; 95% CI, 1.18-1.31). However, because the data were inaccessible, they did not estimate the association between the number of SABs and GD risk.

Induced abortion is a worldwide public health issue. In 57 countries, most of which are in the developing world, induced abortion is prohibited altogether or is allowed only to save the woman’s life, as of the end of 2017.^28^ To date, no study has reported the association of prior induced abortion with GD. Two previous studies^18,19^ have reported the associations of induced abortions with metabolic syndromes or type 2 diabetes. In a cross-sectional survey conducted in 10 375 Chinese women, Xu et al^19^ reported that women with induced abortion history were more likely to have metabolic syndromes (odds ratio, 1.25; 95% CI, 1.06-1.47) than those without a history of induced abortion. In another nationwide prospective study, among 302 669 women from the China Kadoorie Biobank, a history of induced abortion was found to be significantly associated with increased type 2 diabetes risk (hazard ratio, 1.07; 95% CI, 1.02-1.12) later in life, and the association was number dependent.^18^

Understanding the association between SAB and the risk of GD is particularly relevant in China, where the prevalence of GD has been increasing.^29,30^ Our findings suggest that pregnant women with a history of SAB, especially those with a history of recurrent SAB, should attend more antenatal visits to monitor their blood glucose and implement early prevention and intervention (eg, eating more healthfully and doing regular physical activity). Considering the short- and long-term adverse effects of GD on both mothers and their offspring,^4,5,6,7,8^ our findings may also have potential public health implications.

### Limitations

Our study has some limitations. First, the SAB data in medical records were based on self-reported reproductive history. Because abortion is often a sensitive issue, the actual incidence could be underreported, which might result in misclassification of the exposure status and underestimate the strength of the association between SAB and GD. Second, most SABs occurred at home and most of the participants could not recall the exactly date of their SABs. Therefore, we could not include the time between the occur of SAB and the diagnosis of GD in our data analysis. Third, although we attempted to adjust for multiple covariates, residual confounding from unmeasured or unknown confounders remained. Fourth, all participating pregnant women were from one of the largest cities in China, which may limit the generalizability of our findings.

## Conclusions

This cohort study found that a history of SAB was significantly associated with increased risk of GD in subsequent pregnancies. Further research is needed to clarify this association and explore the potential biological mechanisms underlying the association.

## Notes

Supplement. **eTable 1. **Relative Risks (95% Confidence Intervals) for Incident GDM Associated With History of Abortion Among Nulliparous Pregnant Women **eTable 2.** Relative Risks (95% Confidence Intervals) for Incident GDM Associated With History of Abortion Among Pregnant Women Without Family History of Diabetes **eTable 3.** Relative Risks (95% Confidence Intervals) for Incident GDM Associated With History of Abortion Among Pregnant Women Who Conceived Spontaneously
