# Supplementary material for: Association of History of Spontaneous or Induced Abortion With Subsequent Risk of Gestational Diabetes
Source: JAMA Netw Open. 2022 Mar 3;5(3):e220944. doi: 10.1001/jamanetworkopen.2022.0944 (PMC8895257; doi:10.1001/jamanetworkopen.2022.0944)
Supplement: Supplement. — eTable 1. Relative Risks (95% Confidence Intervals) for Incident GDM Associated With History of Abortion Among Nulliparous Pregnant Women eTable 2. Relative Risks (95% Confidence Intervals) for Incident GDM Associated With History of Abortion Among Pregnant Women Without Family History of Diabetes eTable 3. Relative Risks (95% Confidence Intervals) for Incident GDM Associated With History of Abortion Among Pregnant Women Who Conceived Spontaneously [file jamanetwopen-e220944-s001.pdf]

Supplemental Online Content

Zhao Y, Zhao Y, Fan K, Jin L. Association of history of spontaneous or induced abortion with subsequent risk of gestational diabetes. *JAMA Netw Open*. 2022;5(3):e220944. doi:10.1001/jamanetworkopen.2022.0944

eTable 1. Relative Risks (95% Confidence Intervals) for Incident GDM Associated With History of Abortion Among Nulliparous Pregnant Women
eTable 2. Relative Risks (95% Confidence Intervals) for Incident GDM Associated With History of Abortion Among Pregnant Women Without Family History of Diabetes
eTable 3. Relative Risks (95% Confidence Intervals) for Incident GDM Associated With History of Abortion Among Pregnant Women Who Conceived Spontaneously

This supplemental material has been provided by the authors to give readers additional information about their work.

© 2022 Zhao Y et al. *JAMA Network Open.*

eTable 1. Relative Risks (95% Confidence Intervals) for Incident GDM Associated With History of Abortion Among Nulliparous Pregnant Women

	GDM prevalence	Crude model RR (95% CI)	Adjusted model [a] RR (95% CI)
No abortion history	10.4%	1.00 (Reference)	1.00 (Reference)
SAB only	16.0%	1.65 (1.56, 1.74) [b]	1.30 (1.23, 1.38) [b]
Induced abortion	11.0%	1.07 (1.00, 1.15) [c]	1.04 (0.98, 1.12)
Both SAB and induced abortion	14.7%	1.49 (1.32, 1.68) [b]	1.13 (1.00, 1.27) [c]

Abbreviations: SAB, spontaneous abortion; GDM, gestational diabetes mellitus.

[a] Adjusted for maternal age, age at menarche, family diabetes history, the use of ART and pre-pregnancy BMI.

[b] $p < 0.01$, [c] $p < 0.05$.

© 2022 Zhao Y et al. *JAMA Network Open.*

eTable 2. Relative Risks (95% Confidence Intervals) for Incident GDM Associated With History of Abortion Among Pregnant Women Without Family History of Diabetes

	GDM prevalence	Crude model RR (95% CI)	Adjusted model [a] RR (95% CI)
No abortion history	10.3%	1.00 (Reference)	1.00 (Reference)
SAB only	15.2%	1.56 (1.48, 1.64) [b]	1.26 (1.19, 1.33) [b]
Induced abortion	11.7%	1.16 (1.10, 1.22) [b]	1.03 (0.97, 1.09)
Both SAB and induced abortion	15.3%	1.57 (1.44, 1.73) [b]	1.15 (1.05, 1.27) [b]

Abbreviations: SAB, spontaneous abortion; GDM, gestational diabetes mellitus.

[a] Adjusted for maternal age, age at menarche, parity, the use of ART and pre-pregnancy BMI.

[b] $p < 0.01$.

© 2022 Zhao Y et al. *JAMA Network Open.*

eTable 3. Relative Risks (95% Confidence Intervals) for Incident GDM Associated With History of Abortion Among Pregnant Women Who Conceived Spontaneously

	GDM prevalence	Crude model RR (95% CI)	Adjusted model [a] RR (95% CI)
No abortion history	10.6%	1.00 (Reference)	1.00 (Reference)
SAB only	15.5%	1.55 (0.47, 1.63) [b]	1.25 (1.18, 1.32) [b]
Induced abortion	12.2%	1.18 (1.12, 1.24) [b]	1.03 (0.98, 1.09)
Both SAB and induced abortion	15.9%	1.60 (1.46, 1.75) [b]	1.17 (1.06, 1.28) [b]

Abbreviations: SAB, spontaneous abortion; GDM, gestational diabetes mellitus.

[a] Adjusted for maternal age, age at menarche, parity, family diabetes history, and pre-pregnancy BMI.

[b] $p < 0.01$.

© 2022 Zhao Y et al. *JAMA Network Open.*
